# The Religious Fraternities and Brotherhoods of Seville: Servant Leadership in Social Action

**DOI:** 10.3389/fpsyg.2020.00784

**Published:** 2020-04-29

**Authors:** Gonzalo Barrera Blanco, Blanca Martín Ríos, Juan Antonio Senent-De Frutos

**Affiliations:** ^1^Ph.D. Program in Sustainable and Inclusive Developing, Universidad Loyola Andalucía, Sevilla, Spain; ^2^Department of Law, Universidad Loyola Andalucía, Seville, Spain; ^3^Department of Humanities and Philosophy, Universidad Loyola Andalucía, Sevilla, Spain

**Keywords:** religious organizations, volunteer commitment, servant leadership, job satisfaction, implications of laity

## Abstract

The religious fraternities and brotherhoods of Seville, Spain, are among the major agents of the social aid carried out in that city. Knowing the characteristics of the servant leadership that have been established by authors such as Greenleaf (1997), we interviewed several representatives of these institutions to determine if they would meet those characteristics and to what extent they configure their teams in charge of carrying out that social aid. Our aim was to verify, through the Rivera and Santos questionnaire (2015), which has been modified to refer to a legal person, if the characteristics that have been assigned to the concept of leader-server and that normally apply to a natural person can also be identified in such corporations. Moreover, we sought to take the opportunity to investigate this structure of work in social action and identify the common characteristics, if any, that make the fraternities and brotherhoods of Seville different from other private agents fighting against poverty.

## Introduction

In contemporary society, the division of social classes based on financial capacity generates inequality among different sectors of the population. We find groups of people whose financial situation allows them to meet their needs, while other groups’ capacity to do so is either very limited or practically nil.

This wealth/poverty dichotomy is manifested both in comparisons among states and among people within the same city. Therefore, to alleviate differences in financial capacity in regard to meeting needs, social assistance or action programs exist at different geographical levels.

To the traditional division of the first, second and third worlds, the idea of the fourth world has been added, which refers to those within the first world who lack financial capacity.

In Seville, Spain, a first-world city, these situations of need that the State fails to cover through its public administration are addressed through the intervention of private agents whose main activity – or a part of it – is social action. These agents include non-governmental organizations (NGOs) and religious organizations. However, because Spain is a country with deep roots in the Christian tradition, it also has other types of social agents: religious fraternities, brotherhoods and associations of the faithful.

The fact that the researchers are from the city of Seville allows us to better understand these corporations and their profile as social agents dedicated to social action compared to other agents of social intervention, such as NGOs or foundations.

While religious fraternities and brotherhoods are linked to the Catholic Church, they have a different origin than religious congregations. Religious fraternities and brotherhoods arise as associations of lay people centered on devotion to a titular image around which they organize cults for specific purposes. However, they are always linked to a social action and are usually related to a group linked to the corporation, due to their origins in medieval guilds.

According to the majority of authors, such as Moreno (1985), the common purposes shared by all such organizations are to promote the celebration of cults in honor of those for whom they are named, to bring about spiritual improvement among members and to provide charitable care for one another and for the needy in general. Along the same lines, according to other authors such as Valduérteles (2019, p. 2), according to ecclesiastical regulations, their characteristic purposes are the “*promotion of public worship, training of brothers, improvement of society in a Christian spirit, promotion of charity among the brothers (cc. 298 and 301 CIC)*,” thus emphasizing the aspect of the opening the organization to society. Both authors point to charity as the ultimate goal of religious fraternities and brotherhoods, an aspect on which we will focus in this work.

Starting from the notion that social action in a city like Seville represents a social change project involving several actors, such as the State Public Administration and other social agents, we raise the possibility that Greenleaf (1977) idea of servant leadership applies to these lay organizations’ fulfillment of their purpose of exercising social action for the needy in general.

In turn, and assuming that this is indeed true, we are interested in determining the specific characteristics of the teams that lead these organizations.

To do this, in addition to conducting the survey and processing the resulting data, we analyze the following: the concept, evolution and configuration of fraternities and brotherhoods; the translation of the concept of the leader-server to fraternities and brotherhoods as legal persons; the application of the concept of leader-server to fraternities and brotherhoods; the characteristics of the profile of fraternities and brotherhoods; and the new horizons explored through this research.

### Previous Notions

In this research, we address the world of religious fraternities and brotherhoods, a subject that is of great interest at regional and local levels as it is a manifestation of the practically autochthonous popular religiosity of Spain and its former colonies, particularly in Andalusia.

This world, called the “cofrade,” leads us into a social reality that possesses its own vocabulary and idiosyncrasies formed over centuries of history. Therefore, we find it pertinent, before going further into the subject, to discuss a series of previous notions that allow us to clarify some questions that may arise.

The first issue to address is that of language. It is necessary to define and distinguish among concepts and to clarify those terms that cannot be translated or whose translation can lead to error.

We begin with the terms “*hermandades*” and “*cofradías*,” words that could both be translated interchangeably as “brotherhoods.” However, in Spanish, the terms are not the same.

In this work, we prefer to use the term “religious fraternity” for “*hermandad*,” which etymologically approaches but is different from the concept of a “college fraternity” or “fraternity,” highlighting the aspect of religiosity. In addition, we prefer to use the term “brotherhood” for “*cofradía.*” In any case, “*hermandad*” can be used in a broad sense to also refer to brotherhoods, and hence, in this article, we will consider the same use of the concept of “religious fraternity.”

Another construct that we must bear in mind is that of the “*asociaciones de fieles*,” which can be translated as “associations of the faithful” and which constitute a modality within these types of corporations that has an equivalent structure and purpose but occupies a different legal and ecclesiastical category from that of religious fraternities and brotherhoods.

These corporations should not be confused with religious congregations, which are founded by secular people and are usually constituted either only by secular people (such as the Sisters of the Cross), or by secular people and lay people (such as the Jesuits).

Within the internal structure of these corporations, we find the figure of “*Hermano/a Mayor*,” which can be translated as “big brother/sister.” However, we will not use that concept; instead, we shall use “*alcalde*,” or “mayor,” for we believe that this more political term more succinct, since the legal representative of the corporation is democratically elected by the adults in the corporation. We prefer the concept of “mayor” because one of the corporations includes that position, and its powers are identical to those of the “*Hermano Mayor*,” whose position is held by whomever serves as Archbishop of Seville. The term “president” (“*presidente*” in Spanish) would also be valid; however, the term is rarely used in these cases.

The members of the corporation are called “*hermano*” and “*hermana*,” or “brother” and “sister” in English (not to be confused with friars and nuns). However, in Spanish, the plural forms can be “*hermanos*” (only men), “*hermanas*” (only women), or “*hermanos*” (meaning both men and women). This is due to a linguistic norm called the “generic masculine,” whereby the masculine plural of a word can be used to refer to both genders.

The problem is that the term “*hermanos*,” based on the norm of the generic masculine, creates confusion when translated as the neutral English term “siblings,” since we are not talking about a family relationship in legal terms, but rather in spiritual terms (such as “brothers in Christ” and “children of God”). Therefore, it would not be entirely correct to use “siblings.” Hence, we refer to those who belong to the organization as “members” (“*miembros*” in Spanish) or, by applying the Spanish concept of the generic masculine, as “brothers.”

Other concepts that cannot be translated are “*priostía*” (a section of the corporation that is dedicated to the custody and maintenance of objects related to worship), “*rocieras*” (a modality of glory corporations), “*cristíferas*” (relating to avocations of Christ), or “*simpecado*” (flags or banners with the image of a Marian avocation).

Within this particular vocabulary, we must also take into account the “titular image,” “titular of the brotherhood,” or simply, “titular,” which are the figures that exercise a spiritual patronage over the corporation. These may be the Blessed Sacrament, “Cristíferas” avocations, Marian avocations, saints, beati, or “blessed souls” (or souls in purgatory).

These “titulars” are worshiped through the consecrated form, in the case of the Blessed Sacrament, or through paintings, *simpecados*, and, especially, sculptures. The latter are of particular historical/artistic importance in Andalusia and specifically in Seville, where, in addition to being worshiped in church, they are also carried through the streets in processionals.

Most corporations are governed under the General Council of Religious Fraternities and Brotherhoods of Seville, or the Council, which is under the Archdiocese. This institution manages relations among all of the religious fraternities and brotherhoods, the archdiocese, and the public administration. Its functions include facilitating street processionals, promoting the resolution of conflicts between corporations, and distributing the financing they receive from the public administration.

In addition, this institution also carries out its own social action projects and others in conjunction with the religious fraternities and brotherhoods. Actually, in 2002–2004, the Council promoted the carrying out of an investigation into social action, the result of which was reflected in the “White Paper on Social Action”: http://www.hermandades-de-sevilla.org/consejo/accion-social/la-accion-social-conjunta/libro-blanco-de-la-accion-social (accessed in March 2020). However, not all corporations of this type, such as the associations of the faithful, are subject to the authority of the council.

We must clarify that we can identify these corporations based on different criteria and categories. We have opted for the most used method of categorization, which is based on when the corporation ordinarily carries out its main procession; therefore, we distinguish the following categories:

(1) Religious fraternities of Penance: hold processions of penance at the Santa Iglesia Cathedral between Palm Sunday and Easter Sunday;
(2) Religious fraternities of Vespers: hold processions of penance at their location between the Friday of Sorrows and Saturday of Passion Week;
(3) Religious fraternities of Glory: hold processions at a different time than those of the religious fraternities of Vespers and Penance;
(4) Association of the Faithful: holds processions at a different time than those of the religious fraternities of Vespers and Penance; and
(5) Rociera religious fraternities: make a pilgrimage to the Sanctuary of the Virgen del Rocío in Almonte, Huelva, Spain.

The scientific literature on this “cofrade world” is limited, as the existing literature has traditionally focused on historical, theological, legal, and artistic aspects. Treatments in other fields, such as psychology, have been less abundant. In any case, on this topic, we must observe the considerations on the subject of religious fraternities and brotherhoods of Díaz (2011) and his compilation of publications related to the fields of history and law. In addition, although a considerable amount of material exists, it is mostly informative, since thus far not much scientific interest has been generated beyond the local or regional level.

To understand the history of these corporations, it is easiest to turn to informative works. For a more in-depth exploration of their origins, in the absence of scientific publications that can cover all of those in existence, we refer to informative works, particularly the history sections of the websites of each corporation (if they have them), or, failing that, to the summaries on the website of the General Council of Religious Fraternities and Brotherhoods of Seville: http://www.hermandades-de-sevilla.org/hermandades (accessed in September 2019).

Nonetheless, scientific publications do exist and can provide information regarding specific issues, such as when there were still blood-shedding flagellants or brothers (Fernández, 2000; Sánchez, 2000), which, along with other practices, were heavily regulated since 1604 and banned since 1777.

In our case, because social action is the topic being addressed, there is even less reference literature, since this is often created for people from “outside of the cofrade world” who are unfamiliar with it.

However, there happens to be a researcher who specializes in this area: Valduérteles, who is a doctor, university professor and researcher in the field of business, is currently mayor of one of these corporations, and has been publishing about religious fraternities and brotherhoods and their social actions for several years.

Valduérteles is probably the person in Seville who has done the most work in this area, with papers such as *Hermandades para el siglo XXI* (Valduérteles, 2011) and *Informe sobre la caridad en las hermandades de Sevilla* (I, II) (Valduérteles, 2014, 2017a). In recent years, he has published works on brotherhoods outside of the legal, historical or artistic fields, such as *Hermandades 360*° (Valduérteles, 2017b), published by the Cajasol Foundation, among other works.

These publications enjoy wide recognition and have served to raise awareness about the social action of these corporations and how it has developed over time, as we can see in the following article: www.diariodesevilla.es/cofradias-sevilla/Consejo-Accion-Social-Asistencia- Caridad_0_1107189956.html (Accessed in September 2019).

Also, thanks to the “White Paper on Social Action” contributed to the work of Roda (2003): *IV Simposio sobre Hermandades de Sevilla y su Provincia (2003).* Which present the issue of social action highlight the chapter of “*La acción social en las Hermandades: una perspectiva desde la historia y la actualidad*” by De Julios (2003).

For this study, local news articles or specialized articles on the “cofrade” world were of great importance because they offset the lack of information from scientific and current literature, which, from a local point of view, we can directly observe, but would be almost impossible to transmit in full.

This is also true of specific topics mentioned in the article, such as the following:

(1) The current role of women in religious fraternities and brotherhoods; for more on this issue, we recommend the following article: https://sevilla.abc.es/pasionensevilla/actualidad/noticias/la-incorporacion-la- mujer-las-cofradias-ten-anos-after-125220-1521057497.html (accessed September 2019); and
(2) Marginality in Seville, where some of the poorest neighborhoods in Europe are located; information has been published in the media on May 29, 2019, according to the latest edition of “Urban Indicators” by the National Statistics Institute (INE) as part of the European Urban Audit project: https://sevilla.abc.es/sevilla/sevi-poligono-y-pajaritos-sevilla-barrios-mas- poor-espana-201905291330_noticia.html (accessed in September 2019).

Likewise, specialized websites regarding religious fraternities and brotherhoods have also been important. Most of these are local in scope and provide some information that can be extracted.

Regarding social action in particular, to learn about some of these projects, one must visit the websites of the corporations themselves, including the General Council of Religious Fraternities and Brotherhoods of the city of Seville. Some examples can also be found on the website of the Provincial Delegation for Religious Fraternities and Brotherhoods of the Archbishopric of Seville: http://www.hermandades-archi sevilla.org/Accion-Social/index.ph (accessed September 2019).

## Materials and Methodsology

### Materials

We used previous studies in the field of servant leadership as references, such as Greenleaf (1977), Spears (1996); Page and Wong (2000), Marín and Páez (2014); Rivera and Santos (2015), and Blanch et al. (2016).

### Method

Due to both the organizational structure of religious fraternities and brotherhoods and the lack of previous studies on the issue of their social action, we believed that the most suitable option was to conduct interviews based on the questionnaire by Rivera and Santos (2015) to determine whether these groups reflect the characteristics of servant leadership.

Using the same criteria used in this questionnaire, we modified the items to adapt them to the object of study (religious fraternities and brotherhoods), as shown in Table 1.

**TABLE 1:** Survey. First, the interviewee is greeted and introduced to the project. Subsequently, they are asked about their specific position.

Item/Dimension	Question
**Servant leadership**	
Service	The corporation is willing to make sacrifices to provide services to others
Teamwork	When the corporation collaborates with other agents, it values each of the other agents
Setting Objectives	The corporation sets realistic and clear goals
Service	The corporation seeks to serve rather than to be served
Empowerment	It produces great satisfaction within the corporation to help others develop their skills as fully as possible.
Vision	The corporation feels that it is called to do something great for society
Teamwork	When the corporation collaborates with other agents, it looks for ways to make use of the differences among the other agents
Management	The ideas of the corporation are normally accepted by others as useful and effective
Role models	The corporation never asks others to do what it is not willing to do itself
Courage	The corporation has the courage to do things that are morally right, even when doing so may harm it
Integrity	The corporation always keeps its promises and commitments to others
Humility	The corporation does not seek recognition (social renown) nor recompense (financial) for serving others
Care by the Team	The corporation listens actively and receptively to what others have to say
Team discussion	The corporation is willing to have its ideas questioned by people close to it

*To examine servant leadership, they are asked, *Can you tell us to what extent the following statements fit your way of being and thinking?* Response options: Completely disagree, Strongly disagree, Disagree, Neither disagree nor disagree, Agree, Strongly Agree, Completely Agree.*	

To learn about the structure of the teams, the lead-in was as follows: *Please, we would also like to know the following information regarding the team that performs the corporation’s social action*.	

**Item/Dimension**	**Question**
**Composition of the team**	
Permanent physical members	Number of physical persons who permanently make up the team
Occasional physical members	Number of physical persons who occasionally join the team
Gender	Of the permanent members of the team, how many are women and how many are men?
Disability	Based on the legal criteria for disability, how many permanent members of the team qualify as disabled?
Occupation	Occupations of the permanent members of the team (retirees, working, studying, unemployed)
Hiring	How many participate through an employment contract with the corporation?
Remuneration	How many receive financial compensation for their participation?
Commitment	From your perspective, with 0 being very poor and 10 being very good, how would you rate the commitment of the permanent members to the social activity carried out by the corporation?
Work environment	From your perspective, with 0 being very poor and 10 being very good, how would you rate the work environment of the permanent members of the team?
Satisfaction	From your perspective, with 0 being very poor and 10 being very good, how would you rate the team’s satisfaction with the work they do?

*Response options: open.*

Next, a series of technical questions regarding the composition of the teams in charge of social action within the religious fraternities and brotherhoods was incorporated into the questionnaire, as shown in Table 1.

The data were obtained through semi-structured interviews conducted orally. The population selected comprised those occupying the position of mayor or a position equivalent to director of social action (or charity) at the religious fraternities, brotherhoods and associations of the faithful, regardless of the classification of their corporation.

It must be kept in mind that there are several classifications among religious fraternities and brotherhoods, the most common of which distinguishes between “glories” and “penance,” with a subgroup of the latter called “vespers.” Groups with greater recognition are generally those within the “penance” category rather than “vespers,” of which there is a total of 60 in the city of Seville. In total, there are ~126 corporations in Seville governed by the General Council of Religious Fraternities and Brotherhoods.

Of the 30 corporations that participated in this study, at least 24 were penance groups, one was a vespers group, one was an association of the faithful, and four were glories (two of which were “rocieras”). The number of participants is a limitation of our study, because, as the surveys were conducted during the holiday period, it was not possible to locate the responsible members of all of the corporations as their usual activities were suspended.

### Instrument

The first part of the survey follows the model used for Rivera and Santos (2015) questions regarding servant leadership, with the necessary modifications to adapt it to this research.

For the second part of the study, a series of *ad hoc* questions were asked to determine the organizational structure of the team. These questions were asked to clarify, among other aspects, issues such as the number of permanent and occasional members; the employment status of permanent members; gender distribution; participation of individuals with a confirmed disability; characteristics of the employment relationship, if one exists; and the level of satisfaction with the work of the permanent members.

## Results

The results of the investigation are collected in an explanatory legend (Table 2), and the answers are presented in a table (Table 3) showing the responses of the 30 corporations that we were able to survey, which were transformed into a score that varies from “completely disagree = -3” to “completely agree = 3.” In addition, we added a “scoring” column based on the sum of the questions asked of each corporation; a column with the averages of the answers to the last three questions in the survey; the average numbers of women and individuals with disabilities in each corporation; and the average scores, evaluations, and percentages of women and individuals with disabilities.

**TABLE 2:** Legend.

Item	Value	Item	Value	Item	Value	Item	Value	Answer	Value
Service	(1)	Role models	(9)	Volunteers	(17)	Hired	(25)	Completely disagree	−3
Teamwork	(2)	Courage	(10)	Women	(18)	Beneficiaries	(26)	Strongly disagree	−2
Setting Objectives	(3)	Integrity	(H)	Men	(19)	Weight of ratings	(27)	Disagree	−1
Service 2	(4)	Humility	(12)	Disability	(20)	Commitment	(28)	Neither agree nor disagree	0
Empowerment	(5)	Team Care	(13)	Retirees	(21)	Work Environment	(29)	Agree	1
Vision	(6)	Team Discussion	(14)	Worker’s	(22)	Satisfaction	(30)	Strongly agree	2
Teamwork 2	(7)	Score	(15)	Students	(23)	Percentage of Women	(31)	Completely agree	3
Management	(8)	Fixed	(16)	Unemployed	(24)	Percentage with Disabilities	(32)		

**TABLE 3:** Results.

(1)	(2)	(3)	(4)	(5)	(6)	(7)	(8)	(9)	(10)	(11)	(12)	(13)	(14)	(15)	(16)
3	1	3	3	3	2	3	1	3	3	3	3	3	3	37	8
3	2	0	0	3	0	0	1	−1	1	−1	3	2	−3	10	1
3	3	3	3	3	3	2	2	3	3	2	3	3	3	39	3
3	3	3	3	3	3	3	3	3	3	3	3	3	3	42	8
3	3	3	3	2	1	3	2	3	3	3	3	1	0	33	10
3	3	3	3	3	3	3	3	3	3	1	3	1	3	38	6
2	1	2	2	3	2	1	1	3	1	3	3	3	1	28	1
3	3	3	3	3	3	3	*2*	3	3	*2*	3	3	3	40	18
1	1	*2*	*2*	1	1	0	1	1	1	*2*	*2*	3	0	18	5
3	3	*2*	3	3	1	2	3	2	3	1	3	2	3	34	7
3	3	3	3	3	3	3	3	2	3	3	3	3	3	41	10
1	1	1	1	2	2	1	1	1	1	1	1	1	1	16	2
2	1	3	3	3	3	1	2	3	1	3	1	1	−1	26	15
3	1	3	3	3	2	1	2	3	2	2	3	3	1	32	3
3	3	2	3	3	2	3	3	3	2	2	3	3	2	37	35
3	1	3	3	3	3	1	1	2	1	3	3	3	2	32	4
3	1	1	3	3	3	1	1	3	1	3	3	1	1	28	2
0	3	1	2	2	1	2	1	1	2	1	2	3	2	23	6
3	2	2	3	3	3	1	2	2	1	1	3	1	1	28	7
3	3	3	3	3	3	3	3	3	3	3	3	3	3	42	25
1	3	3	3	3	1	1	2	2	3	2	3	3	1	31	10
3	3	3	3	3	1	3	3	1	1	1	3	3	3	34	9
3	1	1	3	3	0	1	1	1	0	3	3	3	0	23	16
3	3	3	3	3	3	3	3	3	2	3	3	3	3	41	10
3	3	3	3	3	1	3	3	3	3	3	3	3	1	38	3
1	1	3	1	3	3	3	1	1	1	1	1	1	1	22	9
2	3	2	3	3	2	2	2	3	2	3	3	2	3	35	4
2	2	2	3	3	1	3	2	1	1	1	3	3	1	28	10
3	3	3	3	3	3	3	1	3	3	3	3	3	3	40	4
3	3	3	3	1	0	1	1	1	0	3	3	2	0	24	10
													Average:	31.33	

(17)	(18)	(19)	(20)	(21)	(22)	(23)	(24)	(25)	(26)	(27)	(28)	(29)	(30)	(31) %	(32) %
45	5	3	1	4	4	0	0	0	0	10.0	10	10	10	62 50	12.50
100	0	1	1	0	0	0	1	0	0	10.0	10	10	10	0.00	100.00
5	1	2	0	0	3	0	0	0	0	9.0	9	9	9	33.33	0.00
6	3	5	0	3	5	0	0	0	0	SO	8	8	8	37.50	0.00
20	7	3	0	5	3	1	1	0	0	8.67	9	9	8	70.00	0.00
30	1	5	0	0	3	0	3	0	0	8.67	8	9	9	16.67	0.00
3	1	0	0	0	1	0	0	0	0	9.00	8	9	10	100.00	0.00
23	15	3	0	0	14	4	0	0	0	10.0	10	10	10	83.33	0.00
3	4	1	0	0	4	1	0	0	0	10.0	10	10	10	80.00	0.00
20	2	5	1	0	4	3	0	0	0	8.33	7	9	9	28.57	14.29
4	7	3	2	2	5	1	2	0	0	10.0	10	10	10	70.00	20.00
5	1	1	0	2	0	0	0	0	0	7.67	8	8	7	50.00	0.00
40	8	7	0	1	6	7	1	0	0	8.33	7	9	9	53.33	0.00
2	1	2	0	0	3	0	0	0	0	9.00	10	10	7	33.33	0.00
10	20	15	2	10	15	6	4	0	0	9.33	9	10	9	57.14	5.71
20	0	4	0	0	4	0	0	0	0	100	10	10	10	000	000
30	0	2	0	0	2	0	0	0	0	10.0	10	10	10	0.00	0.00
47	0	4	2	2	4	0	0	0	0	6.67	7	7	6	0.00	33.33
50	5	2	0	0	7	0	0	0	0	9.00	9	9	9	71.43	0.00
75	16	9	0	0	22	3	0	0	0	9.33	8	10	10	64.00	0.00
6	3	7	0	4	6	0	0	1	0	8.00	8	8	8	30.00	0.00
9	7	2	1	2	7	0	0	0	0	9.67	10	10	9	77.78	11.11
10	9	7	0	10	6	0	0	0	0	10.0	10	10	10	56.25	0.00
80	4	6	1	1	6	3	0	1	0	8.67	8	10	8	40.00	10.00
40	2	1	0	0	3	0	0	0	0	10.0	10	10	10	66.67	0.00
4	2	7	0	3	5	1	0	0	0	9.00	10	7	10	22.22	0.00
20	1	3	0	0	3	1	0	0	0	8.67	9	9	8	25.00	0.00
50	5	5	1	4	5	0	1	0	0	9.33	9	10	9	50.00	10.00
15	0	4	0	0	4	0	0	0	0	6.67	7	7	6	0.00	0.00
30	5	5	0	0	10	0	0	0	0	8.67	10	10	6	50.00	0.00
									Average:	8.99				44.30	7.23

Analysing the data obtained, we found some interesting results, as show the Tables 4 and 5:

**TABLE 4:** Analysis. Part 1.

	Mean	Median	Mode	Standard deviation	Range	Minimum	Maximum	P25	P75
Service	2.5	3	3	0.861033861	3	0	3	2	3
Teamwork	2.23333333	3	3	0.93526074	2	1	3	1	3
Setting objectives	2.4	3	3	085500555	3	0	3	2	3
Service 2	2.66666667	3	3	0.75809804	3	0	3	3	3
Empowerment	2.766666667	3	3	0.568320777	2	1	3	3	3
Vision	1.96666667	2	3	1.06619961	3	0	3	1	3
Teamwork 2	2	2	3	1.050451463	3	0	3	1	3
Management	1.9	2	1	0.84486277	2	1	3	1	3
Role models	2.166666667	3	3	1.053183461	4	−1	3	1	3
Courage	1.9	2	3	1.028892944	3	0	3	1	3
Integrity	2.13333333	2.5	3	1.04166092	4	−1	3	1	3
Humility	2.733333333	3	3	0.63968383	2	1	3	3	3
Team care	24	3	3	085500555	2	1	3	2	3
Team discussion	1.56666667	1.5	3	1.5013404	6	−3	3	1	3
Score	31.3333333	32.5	28	8.36385216	32	10	42	26.5	38
Fixed	8.7	7.5	10	7.320966584	34	1	35	4	10
Volunteers	26.7333333	20	20	25.2776763	98	2	100	0.09375	10
Women	4.5	3	1	502922494	20	0	20	1	6.5
Men	4.13333333	3.5	3	3.03693736	15	0	15	2	5
Disabled	0.4	0	0	0.674664668	2	0	2	0	1
Retirees	1.766666667	0	0	2.725148947	10	0	10	0	2.75
Workers	5.4666667	4	4	4.5692626	22	0	22	6	1
Students	1.03333333	0	0	1 86590707	7	0	7	0	1
Unemployed	0.433333333	0	0	0.971430986	4	0	4	0	0
Hired	0.253708132	0	0	0.253708132	1	0	1	0	0
Beneficiaries	0	0	0	0	0	0	0	0	0
Weight of ratings	8.988888889	9	10	0.944771185	3.333333333	6.666666667	10	8	10
Commitment	8.93333333	9	10	1 11210683	3	7	10	8	10
Work environment	9.23333333	10	10	1.00630198	3	7	10	8	10
Satisfaction	8.8	9	10	1.32352716	4	6	10	8	10
Percentages of fixed members	0.92%	0.26%	0.06%	2.91%	−	0.03%	16.00%	0.19%	0.39%
Percentages of volunteer members	1.48%	0.76%	0.67%	2.33%	−	0.06%	12.00%	0.28%	1.51%

**TABLE 5:** Analysis. Part 2.

Contingency table					
Service	Service 2				Total
	0	1	2	3	
**0**	0	0	1	0	1
**1**	0	2	1	1	4
**2**	0	0	1	3	4
**3**	1	0	0	20	21
**Total**	1	2	3	24	30

χ^2^ Tests					
	Value	df	*P*		
χ^2^	28.4	9	<0.001		
*N*	30				

Contingency table					
Teamwork	Teamwork 2				Total
	0	1	2	3	
**1**	1	7	0	2	10
**2**	1	1	0	1	3
**3**	0	2	4	11	17
**Total**	2	10	4	14	30

χ^2^ Tests					
	Value	df	*P*		
**χ^2^**	16.8	6	0.010		
*N*	30				

Contingency table					
Setting Object-ives	Vision				Total
	0	1	2	3	
**0**	1	0	0	0	1
**1**	1	1	1	1	4
**2**	0	3	3	1	7
**3**	1	4	2	11	IS
**Total**	3	S	6	13	30

χ^2^ Tests					
	Value	df	*P*		
**χ^2^**	17.4	9	0.043		
*N*	30				

Contingency table					
Empowerment	Courage				Total
	0	1	2	3	
**1**	1	1	0	0	2
**2**	0	1	1	1	3
**3**	1	9	4	11	25
**Total**	2	11	5	12	30

χ^2^ Tests					
	Value	df	*P*		
χ^2^	7.92	6	0.239		
*N*	30				

Contingency table					
Management	Role models				Total
	−1	1	2	3	
**1**	1	6	1	4	12
**2**	0	1	2	6	9
**3**	0	1	2	6	9
**Total**	1	8	5	16	30

χ^2^ Tests					
	Value	df	*P*		
χ^2^	7.92	6	0.244		
*N*	30				

Contingency table					
Integrity	Humility				Total
	1	2	3		
**−1**	0	0	1		1
**1**	2	1	5		8
**2**	0	1	5		6
**3**	1	0	14		15
**Total**	3	2	25		30

χ^2^ Tests					
	Value	df	*P*		
**χ^2^**	5.67	6	0.461		
*N*	30				

Contingency table							
Team care	Team discussion						Total
	−3	−1	0	1	2	3	
**1**	0	1	1	4	0	1	7
**2**	1	0	1	0	0	2	4
**3**	0	0	2	5	3	9	19
**Total**	1	1	4	9	3	12	30

χ^2^ Tests							
	Value	df	*P*				
**χ^2^**	16.6	10	0.084				
*N*	30						

Kruskal-Wallis test							
Range							
	1=Commitment; 2=Environment; 3=Satisfaction			*N*		Average range	
Score	1			30		43.77	
	2			30		50.40	
	3			30		42.33	
	Total			90			

Test Statistics							
	Score						
χ^2^	1.839						
gl	2						
Asymptotic Sig.	0.399

(a) Regarding servant leadership:

Although Table 3 presents the specific results obtained for each item, we shall highlight the following results:

(1) The lowest possible rating, “completely disagree” (a value equivalent to-3), was obtained for discussion with the team; integrity and role models received a rating of “disagree” (-1); service, setting objectives, services 2, vision, teamwork 2, and courage received a rating of “neither agree nor disagree” (0); and teamwork, empowerment, direction, humility, and team care received a rating of “agree” (1).
(2) The 75th percentile rating was “completely agree” (3); the 25th percentile ratings for service 2, empowerment, and humility were “completely agree”; in the same percentile, the rating of “strongly agree” (2) was given for service, setting objectives, and team care; and all other variables received a rating of “agree.”
(3) For teamwork, empowerment, humility of management and team care, only ratings of “agree” and “completely agree” were obtained.
(4) The mode of the answers was “completely agree,” except regarding direction, for which it was “agree.”
(5) The rating of “strongly disagree” (-2) was never given.
(6) The median rating, which is equivalent to the 50th percentile, was between “strongly agree” and “completely agree,” with the exception of team management, which still had a median rating higher than “agree.”
(7) The lowest score was 10 points, the mode was 28 points, the average was 31.33 points, the median or 50th percentile was 32.5 points, the 25th percentile was 26.5 points, and the 75th percentile was 38 points of the maximum value of 42.

This work is based on the hypothesis that the organizations analyzed largely conform to the servant leadership model. Therefore, we must assume with respect to the score that the hypothesis is confirmed when a corporation achieves a minimum rating 14 points, that is, the equivalent of an “agree” on each question.

We understood that in order to confirm the hypothesis, given the subjective and personal perceptions of each respondent, we must obtain an average score indicating an affirmative answer, meaning that the sum of the results is at least twice the minimum for the characteristics of servant leadership to indicate ratings of “strongly agree” or a score of 28 points.

Taking as a reference the results of the descriptive study and the data, we obtained another series of results: because the mode is 28 points, the average is 31.33 points and the median is 32.5 points, we could consider the hypothesis confirmed. In addition, we observed that the 25th percentile of the scores (26.5 points) approaches 28; the 50th percentile or median exceeds 28 points, and the 75th percentile vastly exceeds it, with 38 points out of the maximum of 42 possible, which would be equivalent a rating of “completely agree” on the 14 questions.

Analysing the specific data, only 8 of the 30 corporations studied (less than one-third of the sample) had a score below 28 points, and only one obtained less than 14 points. This result does not imply that these corporations approach social action incorrectly but that their practices are perceived as less aligned with the servant leader model; this implies the existence of a different leadership model, which is not necessarily better or worse.

(b) Regarding the leadership team:

In this section, the survey posed some open-ended questions to which the participating corporations could respond freely. We did not expect any specific result, and nor did we propose any hypothesis. This allowed us to obtain information from the people involved in social action in corporations. No information was gathered regarding the number of members in each organization since the same person can be a member (brother) of several corporations and participate in all of them in different ways, which could cause some confusion. Therefore, we focused on specific data regarding social action.

It should be clarified that, when referring to permanent members, we refer to those who hold a permanent position or who always participate voluntarily in social action by directing it. Occasional members or volunteers are those who participate occasionally and do not direct social action.

Most of the survey items referred to the permanent members mentioned first. Based on the data obtained, the following information followed:

(1) No participant obtains economic gain from their collaboration.
(2) Of all the existing personnel in the 30 corporations, there were only two people hired – in different entities – with employment contracts not necessarily linked to social action.
(3) Regarding the employment status of the participants, the majority were employed, followed by retirees, students and, finally, the unemployed.
(4) On average, there were proportionately more women than men, and the maximum ratings were higher among the women. When we analyze the minimum scores and percentiles, we found that when the number of permanent members increases, women outnumber men in groups with more than six women. Therefore, the proportion of women with respect to men was higher in the 75th percentile of the study population, while men outnumbered women in the 50th and 25th percentiles of the study population.
(5) The percentage of permanent members with disabilities was low.

(c) Regarding respondents’ assessments:

Regarding the assessments, because they were also open-ended questions, we did not propose any initial hypothesis. The data indicate that the results were positive, always higher than 5, and the average was equally high. The averages approached 9 out of 10 points, the median was 9 (except for the work environment item, which was 10), and the mode for all of the items was 10. Regarding the percentiles, the 25th percentile score was 8, and 75th percentile was 10.

(d) Regarding the relationships among the servant leadership variables:

To examine these relationships, we created contingency tables for the items that we thought could be dependent using the chi-square test of independence.

As a result, with a 95% confidence level, we matched the variables that seemed conceptually closer to one another:

(1) Service and Service 2:

In this case, the contingency table and the *p*-value indicate that the hypothesis that these two variables are related must be accepted; in fact, the greater the agreement for one of the variables was, the greater the agreement for the other variable was.

(2) Teamwork and Teamwork 2:

According to the contingency table and the *p*-value, the hypothesis that these two variables are related was accepted; in fact, when there was a higher level of agreement for one of them, there was also greater agreement for the other.

(3) Setting Objectives and Vision:

According to the contingency table and the *p*-value, the hypothesis that these two variables are also related was accepted; in fact, the greater the agreement for one was, the greater the agreement was for the other.

(4) Empowerment and Courage:

In this case, we can deduce from the contingency table and the *p*-value that we must accept the hypothesis that these two variables are not related and are independent.

(5) Management and Role Models:

Similarly, we can determine from the contingency table and the *p*-value that we must accept the hypothesis that the two variables are not related and are independent.

(6) Integrity and Humility:

Based on the contingency table and the *p*-value, the hypothesis that these two variables are not related, but rather are independent, was accepted.

(7) Team Care and Team Discussion:

Similarly, it follows from the contingency table and the *p*-value that the hypothesis that these two variables are not related must be accepted, as they are also independent.

(e) Regarding the relationship between participation and the number of brothers in each corporation:

For this part of the study, we considered the relationship between the number of brothers in each organization and the form of participation: fixed or voluntary.

For data protection reasons, the values of these variables cannot be shown in the tables, as some corporations can be identified by their membership numbers. However, the results of the descriptive study can be provided because, although they are of interest to the study, they are based on data that cannot be used to identify participating organizations.

During this study, the question of the possible relationship between the number of members (brothers) and their participation in social action was raised. Actual data on members are difficult to access and validate as they are subject to constant changes; hence, we cannot guarantee the use of a system of authentication or common verification.

For this reason, we used an estimate of the number of members published annually for all penance and rocieras corporations as a reference, however, this is approximate data because the number of members varies depending on how many join and leave. Estimates were published for 27 of the 30 corporations with which we worked, and we also have approximate data regarding the number of members in one of the remaining three that was provided by the corporation itself during the interview.

For the corporations for which we could not obtain an estimate, we chose to assign an approximate value based on their characteristics and the number of estimated members (maximum and minimum) in the rest of the corporations.

The consequences of this decision were as follows:

Not taking into account the corporations without published membership estimates, the Pearson’s coefficient of variation was −0.09, which does not reliability indicate a linear progression.

When all the estimated data were used, Pearson’s coefficient of variation was 0.8, which indicates the possibility of a linear progression. However, when we added the data for the corporation that provided an estimated number of brothers, which was the lowest estimate among those collected, Pearson’s coefficient of variation remained at 0.8. This implies that the data would have a distribution close to normal for these variables, and hence, when one of them is known, the results for the other are more easily predicted.

In corporations with fewer members, the percentage of participation, both fixed and voluntary, is much higher because proportionally more group members participate.

On the other hand, when we increased the approximate number of members of corporations for which we estimated membership numbers, Pearson’s coefficient of variation was also maintained at ∼0.8.

In this case, we observed in the descriptive analysis that the maximum percentages of permanent members who participate exceeds the maximum percentages of voluntary members who do so, although the results indicate that there were more volunteers than permanent members. This is because the group of volunteers is normally larger than that of organizers, however, in Table 3, we can observe some exceptions, which are affected by the number of members (brothers) in the corporation.

(f) Regarding the ratings:

We also analyzed the valuations using the Kruskal-Wallis test by ranks, for which we had to use a different distribution of the data.

As a null hypothesis of this contrast, we propose that the distribution of the valuations made by the different corporations is on average the same as for the last three issues raised.

The result shows that the *p*-value (or asymptotic sig.) is 0.399, higher than the usual level of significance of 0.05; thus, there is insufficient evidence to reject the null hypothesis as the distribution of the assessments is the same on average.

## Discussion

### Concept, Evolution, and Configuration of Religious Fraternities and Brotherhoods

To better understand the importance of religious fraternities and brotherhoods, we must bear in mind that we are talking about organizations with a corporate, democratic structure regulated by both canonic law and civil law, in which diocesan regulations also have a very important and direct role (Ribelot, 2000). Some corporations have more than 600 years of history and have been dedicated to social action in the city of Seville since their inception (Moreno, 1997).

Different divisions and classifications of these organizations exist, including those by Moreno (1985, p. 41–69) and Sánchez (2000), however, other criteria can be added, such as brotherhoods based on guild, ethnicity, class, neighborhood, open or closed nature, penitence, glory or sacrament, etc. It should be added that one cannot always identify a religious fraternity with a brotherhood since, in general, not all brotherhoods have the rank of a religious fraternity.

Religious fraternities and brotherhoods, especially penitential ones, with their participation in and organization of Holy Week, are part of popular religiosity (Fernández, 2000). The idea of the penance way in Holy Week (Estrada, 2000, p. 213–235) is undoubtedly the most well-known aspect of most of these corporations, and their other dimensions and purposes are often unknown.

The evolution of religious fraternities and brotherhoods (Sánchez, 2000) and Holy Week in Seville is part of the history of the city itself and has gone through different stages of boom and decline (Moreno, 2000). In fact, the adjective “popular” allows us to state that in some cases, these organizations have transcended their religious aspect to acquire a certain festive character, though they are always linked to ethical, social and traditional issues.

Many of the peculiarities, both current and historical, of these corporations have also been studied in the field of psychology. Such particular issues include the reasons for participation, class differences, different forms of participation between men and women, or the matrilineal affiliation (by maternal line). These characteristics are common in many religious fraternities because they also reflect social evolution (Moreno, 1985).

The legal representative of the corporation is generally called the mayor (Ribelot, 2000), while the rest of the group are known as members (brothers). Alongside the mayor, there is a managing body called the governing board, whose members are democratically elected through universal suffrage among the adult members. Members of note include a deputy/counselor of charity, who is in charge of carrying out the corporation’s charity works. These individuals are preferentially selected to constitute the population chosen for the survey.

### Translation of the Servant Leader Concept to Religious Fraternities and Brotherhoods as a Legal Person

Of great importance in this area is the study by Marín and Páez (2014), which applies the theory of servant leadership to organizations and defines some of its characteristics, focusing on entities that follow a business model with workers and clients. In this study, however, we examine religious corporations with volunteers and beneficiaries of social action, a perspective that has not been analyzed to date. This justifies the need for this study to publicize a previously unknown aspect, which allows the perspective of servant leadership to be applied to an organizational model whose purpose is service to society, rather than economic benefit.

Thus, we can compare the characteristics that identify servant leadership, with an understanding of both the natural person and the legal person.

Regarding the natural person as a servant leader, we note the characteristics highlighted in the study by Villalba (2018): listening, empathy, healing, awareness, persuasion, conceptualization, foresight, administration, commitment to people’s growth, and community building. In addition, as Villalba (2018, p. 25) also states: “*This type of leadership does not seek recognition, since it believes that recognition comes without having been sought, and instead of monopolizing it and boasting, the leader always shares it with everyone involved, thanks to which others feel appreciated [and] have the desire to continue contributing to the work they perform (Hernández, 2017*).”

Regarding the legal entity as a servant leader, we follow the proposal of Marín and Páez (2014), which highlights the following characteristics: “*Caring for people, the most capable and the least capable serving each other, is the cornerstone on which a good society is built. Considering that, until recently, care was to a large extent given from person to person, now the vast majority is mediated through institutions that are often large, complex, powerful, and impersonal; not always competent; and sometimes corrupt. To build a better, more just and loving society, one that can offer better opportunities to its members, the best way to do so is by increasing both the ability to serve and the proper performance as servants of the main existing institutions through new regenerative forces operating from within them (Anderson, 2008: 6)*” (Marín and Páez, 2014, p. 112).

We cannot lose sight of the paradox of the “lead to serve” paradigm, which arises from the leader’s natural predisposition not to identify him or herself as a “selfish leader” (Marín, 2015, p. 15). In these cases, the assumption is that the mentality of the corporation itself is the search for service itself. Along these lines, we see that, indeed, “*servant leadership is paradoxical because it is difficult to understand that one must lead to serve, not to be served. The servant leader chooses to serve first, and then lead, as a way to expand the spectrum of service to individuals and institutions*” (Marín, 2010, p. 34). These types of corporations seek to serve and expand their service to more people.

The concept of the natural person within servant leadership complements the vision of the characteristics that we have seen in the legal person. This constitutes a reality within these corporations since, given their origins, any new corporation that is created is linked to a certain social action related to the cult and the spiritual training of the members, although this can be modified over time.

We must not lose sight of the fact that many of these corporations have their historical origins in guilds, or of the influence of the Catholic Church in Spain and how it generated guilds (as occurred with freed black slaves or Romani) as ways of attending to the needs of their members and others close to that environment by establishing a legal association within the Church to raise money without relying on begging and to foster trust through the spirit of charity as a Christian virtue.

An example of this is in the guild, or group, origins of religious fraternities linked to potters, tailors, sailors and port workers, workers at the Royal Tobacco Factory of Seville, etc., and more modern groups such as the university, the Civil Guard and other military and police bodies, students at religious schools, and people in other circumstances, such as freed black slaves or Romani.

These groups were moved by the needs of the people around them, especially when they ceased to be productive, which prompted personal and family crises. The groups sought a way to assist those in need, either by promoting the construction of hospitals and hospices where members could end their days or by directly providing financial assistance to their neediest members. With this objective, the so-called “charity exchange” was created to assist widows or orphans and to exercise guardianship and legal representation.

The characteristics of the corporations have since been modified because their members are now associated with them mainly by family tradition, marriage, friendship with a member, or devotional matters.

In the same way, the social action performed by these corporations has evolved. The emergence of the modern state, which assumes a governmental responsibility aimed at achieving the characteristics of the welfare state, has caused social action to move toward other purposes or to complement the public administration by addressing objectives that it does not. Of particular importance is the elimination of dependence on the group as a requirement for beneficiaries of aid from a corporation; this change has resulted in the opening of social action to almost anyone who requests it, with the exception of projects created for specific populations (such as single mothers or people with certain diseases or addictions).

In any case, the corporations continue to assume the objective of improving society by taking an ethical and charitable approach to those in need, regardless of whether they are members of the organization. We observe the characteristics previously proposed by Villalba (2018) and Marín and Páez (2014) in the corporations’ perceptions of the purpose of their social action. Thus, it is noted that they seek to positively influence society and individuals, acting as their servants in situations of material need but without seeking direct recognition, since they understand that they serve society as part of their own institutional nature. Hence, in our survey, we did not find responses below the “agree” value on questions regarding humility.

### Application of the Servant Leader Concept to Religious Fraternities and Brotherhoods

Taking into account these approaches to servant leadership and analysing the characteristics of religious fraternities and brotherhoods as well as the results of the surveys, we can conclude that this type of leadership exists both in these corporations and in their relationships with their teams of volunteer workers, most of whom are laypeople, in the development of their social action.

Particularly striking is the fact that this leadership model is not one that was implemented recently; rather, it has been a defining characteristic of these corporations for several centuries, in some cases.

However, it is necessary to take into account that this study is based on the respondents’ own perceptions, and we could not determine with total accuracy when this specific leadership model was used in each corporation or when a different one was used. However, we understand that based on the perspective and purpose of corporations whose vision is associated with the charitable nature of the corporation, this leadership model applies.

The corporations surveyed were unaware of the characteristics of this type of leadership and responded critically and sincerely as requested; as a result, the analysis of the data revealed positive responses regarding the characteristics of servant leadership. Therefore, we can state that the leadership of these corporations, for the most part, very closely fits the profile of the servant leader.

In fact, we believe that the variety of responses reflects the diversity of each corporation and the respondents and that both the size of the corporation and the amount of time the person surveyed had been responsible for the social action of the entity influenced the results.

Conducting the survey allowed us to verify that the general perceptions of those responsible for the social action of religious fraternities and brotherhoods in Seville are very close to the characteristics of the servant leader model, thus facilitating the comparison of these corporations with the servant leadership model.

### Characteristics of the Workers in Religious Fraternities and Brotherhoods

The analysis of the results of the second part of the survey demonstrates the existence of some very interesting characteristics of these corporations, at least in the entities surveyed:

A very striking result was the number of participants, both permanent, ranging from 1 to 35 people depending on the corporation (the mode being 10, the median 7.5 and the total average 8.7 people), and volunteers, which fluctuated from 2 to 100 people (the mode and median were 20, and the average was 26.73 people).

The incompatibility of positions is a common characteristic of these organizations; thus, those with an official position in one council usually may not hold a position in another corporation (depend on the internal rules). Among the 30 corporations analyzed, there were ∼261 people (a figure resulting from summing the values for all corporations) who permanently work for free for social action in Seville, a city that has a population of close to 700,000 inhabitants and includes some of the poorest neighborhoods in Europe.

This figure for permanent staff contrasts with the percentage of members in each corporation, which may seem low. In relation to this, however, it must be taken into account that while posts are incompatible, membership in different religious fraternities and brotherhoods is not, and hence it is common for people belong to several different corporations but not participate equally in all of them.

Another influencing factor is that at present, not all members are residents of the city or the geographical setting of the corporation. Another factor is differences in ages and personal occupations, since the members of a corporation can participate in other sections (for example, in *priostía*) or as assistants and collaborators in social action projects (attending activities that are organized to raise funds for the charity exchange) since social action is one form of participation within corporations, but not the only one. Similarly, involvement is also based on personal factors, since one may participate on the basis of faith or for reasons of culture or tradition.

In any case, the number of volunteers depends on the size of the corporation in terms of its number of members; there are very significant differences in this area (entities may include 50, 2,000, 10,000 or more people), and size has a decisive influence on a corporation’s financial and personnel capacities.

Another interesting aspect is the presence of women among permanent workers, a fact that is very clear in the proportions of women among the participants. The results indicate that, based the presence of six women in permanent positions, the representation of women will always be superior to that of men. However, in a greater number of cases, no women were involved in the social action of the corporation, compared to a single corporation in which no men were involved in social action.

Although women’s presence in some brotherhoods has traditionally been minor – or even null – until the end of the 20th century and the beginning of the 21st century, the results of the present study reflect the success of this change, which was propitiated so that women could also participate in processions of penance and serve on the governing boards of corporations, becoming involved in decision-making and management.

On the other hand, the participation of permanent members with disabilities was not exactly high, taking into account the existence of various types of disabilities and the fact that participation is free and voluntary. Quite striking was the presence of people with a disability that prevents them from working who altruistically dedicate their time to managing projects to help others; this contrasts with the cases of NGOs, foundations and companies that specifically hire people with disabilities, which can provide tax benefits.

This also contrasts with the high number of people in fixed and voluntary positions in the corporations surveyed who were employed. Especially striking was the number of women, who, in addition to working and caring for their family (specifically, their children and parents), find time to devote to project management to meet the needs of others, voluntarily and free of charge.

### New Horizons in Research

In conducting this study and examining the results, we verified the difficulty of addressing these types of questions in the absence of related scientific research, Valduérteles being the exception in the area of charity.

For those researchers who deal with the issue of inclusive and sustainable development, the possibility of learning about realities and research that offer different approaches to problems by trying to bring about improvements in society becomes especially important. This study proposes a different model for addressing the widespread manifestations of poverty and situations of social marginality, which underscore the importance of publicizing this model of organizations based in Spain and, specifically, in the city of Seville.

With this study, we wish to encourage research and analysis on what may be the least-known aspects of these institutions outside of the traditionally studied areas and those of more local interest, such as the fields of religious (given their link with faith), history (given their continued presence over time), the law (given their legal singularities), ethnological (given their status as cultural manifestations) and art (in terms of their artistic heritage).

We also believe that it would be interesting to deepen knowledge about this reality by comparing the results that we have obtained from the profile of the servant leader in these corporations with the profile of servant leadership among natural persons, other classes of legal persons (such as NGOs, foundations, religious congregations, etc.), or similar corporations in other geographical locations.

Finally, and from the perspective of inclusive and sustainable development, such study would allow comparisons with organizational models other than NGOs for religious congregations that seek to improve their interventions and achieve greater success in reducing poverty and integrating marginalized populations through private cooperation.

## Conclusion

Given the findings of the study, we consider our main hypothesis to be valid: the religious fraternities and brotherhoods of the city of Seville, as private agents engaged in social action, operate with a profile very close to that of a servant leader, although these entities are legal persons.

Those surveyed demonstrated very natural responses to the types of questions posed, demonstrating a high degree of understanding and requesting only specific clarifications with the intention of adapting their response to questions that seemed ambiguous.

An example of this was that regarding the vision parameter, some respondents asked what was meant by “something big” since, based on their responsibility and perception, they accepted how limited a social action project can be with respect to number of people in need, the characteristics of the needs they could cover as an organization and their own resources.

Also noted was the fact that in the corporations’ budgets, charity or social action accounts for the most significant fixed portion of income and expenses, highlighting the fundamental nature of social action for these corporations above other expenses.

Finally, we cannot finish without mentioning three important cross-cutting issues.

The first is the importance to a corporation of its titular image and motto. Titular images always reflect aspects and virtues of the Christian faith to encourage greater spiritual and personal growth among members, promoting ethical values among subjects in order to improve society through their particular participation in the corporation.

To reinforce that idea, corporations have a shield and a motto. Specifically, the motto serves to reinforce the spiritual mission and social action of corporations.

Consequently, the titular image, which historically is of a catechetical nature, and the motto, in its mission of promoting charity as a virtue among members, are what provide meaning to the membership of the corporation outside of the traditional or legal arena.

The second issue lies in the differences in the concepts of charity, solidarity and social action. In the sphere of corporations, the term “charity” is usually preferred; however, as Valduérteles (2019) states, charity is a theological virtue that lies in the person and is not quantifiable, solidarity is a human virtue that lies in the person and is not quantifiable, and social action is an activity that lies in the religious fraternity and is quantifiable. We sought to make this distinction clear in the article.

The final issue relates to the content of the social action of religious fraternities and brotherhoods. Throughout the article, we have discussed the existence of social action among these entities, but without specifying its exact content.

The aim of this work is not to analyze the specific projects of the different corporations to examine whether they meet their objectives, how many people they help, their budgets, etc. However, for the purpose of this study, it is important to at least provide examples of this social action.

Social action, as we said, was historically linked to assistance for persons belonging to a guild or collective; today, however, such assistance is provided by the public administration through free public services and financial assistance. However, the assistance provided by the state is not always sufficient or immediate, and in such cases, corporations play an essential role, either through assistance or mediation.

Corporations engage in very different forms of social action, which are conditioned by both the size of the entity and the circumstances. Among the perhaps countless number of actions, we can name the following interventions performed by corporations: paying occasional bills and invoices; obtaining services for free or at a better price through members; contributing funds or goods to social commissaries that distribute them to those who need them; distributing clothes, school supplies or toys; creating specialized foundations and centers for certain situations; creating specialized treatment centers; creating scholarships and study grants; cooperating with other agents for social purposes; helping people find employment; accompanying people who are alone; organizing summer camps for children; welcoming children from needy countries during the summer; helping convents and small religious communities to offset their external dependence or cover some of their needs, etc.

On the other hand, after interviewing the respondents and speaking with Valduérteles, who has researched the aspect of charity among these corporations, we must stress the following: beyond the numbers, money or goods that may be provided (which, at the discretion of corporations, is not fully known), there are other benefits that cannot be quantified, such as the happiness of those who feel accompanied, heard, or understood; those who feel that they have recovered lost dignity; those who are released from a debt or situation from which they see no exit; the happiness of those who have participated in a project that has served society; and the happiness of children who can maintain their childhood and enthusiasm despite their needs and illnesses.

## Data Availability Statement

All datasets generated for this study are included in the article/supplementary material.

## Ethics Statement

The studies involving human participants were reviewed and approved by Comité de Ética Universidad Loyola Andalucía. The participants provided their written informed consent to participate in this study.

## Author Contributions

GB was responsible for fieldwork, statistics, and writing part of the article, with contributions in the area of religious fraternities and brotherhoods. BM was responsible for writing most of the article, with contributions in the area of servant leadership. JS was responsible for directing the project, coordinating and ensuring the coherence of the research, and the technical aspects of writing the article.

## Conflict of Interest

The authors declare that the research was conducted in the absence of any commercial or financial relationships that could be construed as a potential conflict of interest.

## References

[B1] Anderson J. (2008). *The Writings of Robert K. Greenleaf: An Interpretive Analysis and the Future of Servant Leadership*. Virginia Beach, VA: School of Global Leadership & Entrepreneurship, Regent University.

[B2] Blanch J. Gil F. Antino M. Y. Rodríguez-Muñoz A. (2016). Modelos de liderazgo positivos: marco teórico y líneas de investigación. *Papeles Psicól.* 37 170–176.

[B3] De Julios A. (2003). “La acción social en las Hermandades: una perspectiva desde la historia y la actualidad,” in *Proceedings of the IV Simposio de Hermandades de Sevilla y su provincia, Guadalquivir S.L. Ediciones*, ed. Roda Peña J. (Spain: Fundación Cruzcampo), 15–30.

[B4] Díaz B. (2011). La investigación histórica y jurídica de las cofradías y hermandades de pasión en Andalucía. *Foro Nueva Época* 14 195–222.

[B5] Estrada J. A. (2000). “La Pasión según Sevilla: algunas claves teológicas,” in *Religiosidad Popular Sevillana*, ed. Hurtado J. (Spain: Universidad de Sevilla/Área de cultura-Ayuntamiento de Sevilla).

[B6] Fernández E. (2000). “La religiosidad popular sevillana en sus manifestaciones de culto externo,” in *Religiosidad Popular Sevillana*, ed. Hurtado J. (Spain: Universidad de Sevilla/Área de cultura-Ayuntamiento de Sevilla).

[B7] Greenleaf R. K. (1997). *Servant Leadership: A Journey into the Nature of Legitimate Power and Greatness.* Mahwah, NJ: Paulist Press.

[B8] Hernández J. D. (2017). *Liderazgo Moral.* Santa Cruz, Bolivia: Editorial Gemas.

[B9] Marín G. C. W. (2010). *Liderazgo Servidor. Hacia un Nuevo Enfoque en el Liderazgo.* Antioquia: Corporación Universitaria Adventista, 31–35.

[B10] Marín G. C. W. (2015). *Reflexionando en Liderazgo: ¿Paradigma? O ¿Paradoja?.* Antioquia: Corporación Universitaria Adventista, 14–17.

[B11] Marín G. C. W. Páez C. D. Y. (2014). Aplicación del liderazgo servidor en las organizaciones. *Sotavento M.B.A.* 23 108–129.

[B12] Moreno I. (1985). *Cofradías y Hermandades Andaluzas. Estructura, simbolismo e identidad Biblioteca de la cultura andaluza*. Sevilla, Spain: Editoriales andaluzas unidas S.A.

[B13] Moreno I. (1997). *La Antigua Hermandad de los Negros de Sevilla. Etnicidad, Poder y Sociedad en* 600 años de historia. Spain: Universidad de Sevilla/Consejería de Cultura- Junta de Andalucía.

[B14] Moreno I. (2000). “Identificaciones colectivas, modernidad y cultura andaluza: la Semana Santa de Sevilla en la era de la globalización,” in *Religiosidad Popular Sevillana*, ed. Hurtado J. (Spain: Universidad de Sevilla/Área de cultura-Ayuntamiento de Sevilla).

[B15] Page D. Wong T. P. (2000). *A Conceptual Framework for Measuring Servant Leadership. The Human Factor in Shaping the Course of History and Development.* Lanham, MD: University Press of America.

[B16] Ribelot A. (2000). *El derecho de las cofradías de Sevilla: Materiales para el estudio del Derecho Canónico y las Hermandades.* Sevilla, Spain: Marsay Ediciones.

[B17] Rivera R. Santos D. (2015). *El Perfil de los Futuros Emprendedores Sociales: Competencias y Estilos de Vida.* Milton Park: Taylor & Francis, 13–28.

[B18] Roda J. (2003). *IV Simposio sobre Hermandades de Sevilla y su Provincia. Guadalquivir S.L.* Spain: Fundación Cruzcampo.

[B19] Sánchez J. (2000). “La evolución de las cofradías de Semana Santa en la actual diócesis de Sevilla desde sus fundaciones hasta nuestros días,” in *Religiosidad Popular Sevillana*, ed. Hurtado J. (Spain: Universidad de Sevilla/Área de cultura-Ayuntamiento de Sevilla).

[B20] Spears L. (1996). Reflections on Robert K. Greenleaf and servant-leadership. *Leadersh. Organ. Dev. J.* 17 33–35. 10.18549/PharmPract.2018.02.1192 30023031 PMC6041211

[B21] Valduérteles I. (2011). *Hermandades Para el Siglo XXI.* London: IIAP.

[B22] Valduérteles I. (2014). *Informe Sobre la Caridad en las Hermandades de Sevilla Ed.* Baltimore: Fundación CRS.

[B23] Valduérteles I. (2017a). *Hermandades 360°.* Spain: Fundación Cajasol.

[B24] Valduérteles I. (2017b). *Informe Sobre la Caridad en las Hermandades de Sevilla (II) Ed.* Baltimore: Fundación CRS.

[B25] Valduérteles I. (2019). “Caridad en las hermandades,” in *Proceedings of the Seminario: Caridad en las Hermandades* (Sevilla, Spain: Ed. Instituto de Investigación Aplicado a las Pymes).

[B26] Villalba P. A. (2018). *Diseño de un Programa de Desarrollo de Competencias de Liderazgo de Servicio en los Funcionarios del Ministerio del Trabajo. En el periodo 2017, Matriz Quito.* Disertación previa a la obtención del título de Psicóloga Organizacional, Pontificia Universidad Católica del Ecuador, Quito.

